# Reply to Jue et al. Overcoming Understaging and Undergrading in Upper Tract Urothelial Carcinoma. Comment on “Ghoreifi et al. Modern Kidney-Sparing Management of Upper Tract Urothelial Carcinoma. *Cancers* 2023, *15*, 4495”

**DOI:** 10.3390/cancers16051005

**Published:** 2024-02-29

**Authors:** Alireza Ghoreifi, Reza Sari Motlagh, Gerhard Fuchs

**Affiliations:** 1 Institute of Urology, University of Southern California, Los Angeles, CA 90089, USA; gerhard.fuchs@med.usc.edu; 2 Urology Department, University of Vienna, 1090 Vienna, Austria; reza.sarimotlagh@meduniwien.ac.at

We appreciate the comments made by Jue et al. on our manuscript ‘Modern kidney-sparing management of upper tract urothelial carcinoma (UTUC)’ [1]. The authors judiciously highlighted the limitations of ureteroscopic biopsy in patients with UTUC. Despite certain risks like undergrading and understaging, a substantial correlation between tumor grade upon ureteroscopic biopsy and the final pathology exists, especially for high-grade tumors. In addition, the detection of clinical high-grade tumors and subepithelial connective tissue invasion in ureteroscopic biopsy has demonstrated a moderate and strong correlation with invasive UTUC, respectively [2]. Hence, the recent American Urological Association (AUA) guidelines strongly recommend the diagnostic ureteroscopy and biopsy of any identified lesion in patients with suspected UTUC [3]. It is worth noting that preoperative clinical staging remains challenging to assess given the limitations of ureteroscopy and other currently available diagnostic modalities, including cross-sectional imaging and urine cytology. Therefore, both AUA and European Association of Urology (EAU) guidelines recommend a risk stratification model to classify patients as being at low- or high-risk of invasive disease (pT2 or greater) [3,4]. This model will be further used for decision making in systemic and surgical treatments, favoring nephron-sparing therapies in patients with low-risk disease.

To address the limitations of ureteroscopic biopsy for proper tissue sampling, Jue et al. proposed en-block enucleation as a potential solution. While the feasibility of this approach has been demonstrated in a few case reports [5,6], offering the advantage of enhanced histopathological information, its indications and oncological safety remain to be determined. Technical considerations to avoid perforation and identifying the appropriate margin of resection are major challenges of this procedure, especially in ureteral tumors. Furthermore, the feasibility and efficacy of this approach in multifocal and flat lesions is not clear. 

Despite the crucial role of ureteroscopic biopsy in the diagnosis and risk stratification of patients with UTUC, it significantly increases the risk of intravesical recurrence following radical nephroureterectomy [7]. Using a ureteral access sheath has the potential to mitigate this risk by reducing pelvicalyceal hydrostatic pressure; however, the advantages of this approach require further validation [8]. Considering these caveats, efforts have been made in recent years to explore novel diagnostic and prognostic modalities. Recent studies have shown promising results in this context, particularly with the use of non-invasive modalities such as blood- and urine-based genomic and epigenetic biomarkers [9,10]. Future investigations are warranted to determine the optimal approach for the diagnosis of patients with UTUC. 
